# Vascular Relaxation Induced by C-Type Natriuretic Peptide Involves the Ca^2+^/NO-Synthase/NO Pathway

**DOI:** 10.1371/journal.pone.0095446

**Published:** 2014-05-01

**Authors:** Fernanda A. Andrade, Carolina B. A. Restini, Marcella D. Grando, Leandra N. Z. Ramalho, Lusiane M. Bendhack

**Affiliations:** 1 Department of Pharmacology, School of Medicine of Ribeirão Preto - University of São Paulo, São Paulo, Brasil; 2 Department of Medicine – University of Ribeirão Preto, Ribeirão Preto, Brasil; 3 Department of Physics and Chemistry, Faculty of Pharmaceutical Sciences of Ribeirão Preto - University of São Paulo, São Paulo, Brasil; 4 Department of Cellular and Molecular Pathology, School of Medicine of Ribeirão Preto - University of São Paulo, São Paulo, Brasil; University of Southampton, United Kingdom

## Abstract

**Aims:**

C-type natriuretic peptide (CNP) and nitric oxide (NO) are endothelium-derived factors that play important roles in the regulation of vascular tone and arterial blood pressure. We hypothesized that NO produced by the endothelial NO-synthase (NOS-3) contributes to the relaxation induced by CNP in isolated rat aorta via activation of endothelial NPR-C receptor. Therefore, the aim of this study was to investigate the putative contribution of NO through NPR-C activation in the CNP induced relaxation in isolated conductance artery.

**Main Methods:**

Concentration-effect curves for CNP were constructed in aortic rings isolated from rats. Confocal microscopy was used to analyze the cytosolic calcium mobilization induced by CNP. The phosphorylation of the residue Ser^1177^ of NOS was analyzed by Western blot and the expression and localization of NPR-C receptors was analyzed by immunohistochemistry.

**Key Findings:**

CNP was less potent in inducing relaxation in denuded endothelium aortic rings than in intact ones. L-NAME attenuated the potency of CNP and similar results were obtained in the presence of hydroxocobalamin, an intracellular NO^0^ scavenger. CNP did not change the phosphorylation of Ser^1177^, the activation site of NOS-3, when compared with control. The addition of CNP produced an increase in [Ca^2+^]_c_ in endothelial cells and a decrease in [Ca^2+^]_c_ in vascular smooth muscle cells. The NPR-C-receptors are expressed in endothelial and adventitial rat aortas.

**Significance:**

These results suggest that CNP-induced relaxation in intact aorta isolated from rats involves NO production due to [Ca^2+^]_c_ increase in endothelial cells possibly through NPR-C activation expressed in these cells. The present study provides a breakthrough in the understanding of the close relationship between the vascular actions of nitric oxide and CNP.

## Introduction

C-type natriuretic peptide (CNP) and nitric oxide (NO) act as synergic elements in the regulation of vascular tone and arterial blood pressure, thereby playing an important role in the maintenance of cardiovascular homeostasis [1], [2]. CNP belongs to a family of natriuretic peptides, which also includes atrial natriuretic peptide (ANP), brain natriuretic peptide (BNP) and Urodilatin. CNP is abundantly present in vascular endothelial cells [3] but it is also expressed in other tissues [4]. Due to the fact that CNP is an important vasodilator with few renal actions, it has been suggested that this peptide has a function as a paracrine mediator to regulate vascular smooth muscle tone and blood flow [3], [5].

Physiological effects of CNP are mainly mediated through its high affinity binding to membrane-integrated natriuretic peptide receptors NPR-B and NPR-C, which are strongly expressed in venous tissue, aortic smooth muscle and aortic endothelial cells [6], [7]. The activation of NPR-B by CNP leads to an increase in cytosolic guanosine 3′,5′-cyclic monophosphate (cGMP) which mediates cellular responses [7]. On the other hand, the NPR-C that had been primarily considered as a clearance receptor is devoid of guanylyl cyclase activity and its activation by CNP can result in adenylyl cyclase inhibition through an inhibitory guanine nucleotide regulatory (G_i_) and/or phospholipase C (PLC) activation [8], [9].

Besides, it is known that the synthesis of nitric oxide in the vascular endothelial cells also plays a key role in the regulation of vascular tone and arterial blood pressure [10]. It is well documented that endothelial production of NO is regulated by endothelial NO synthase (NOS-3) activation in response to an increase in cytosolic Ca^2+^ concentration ([Ca^2+^]_c_) [1], [11], [12]. However, the activation of NOS-3 can also occur in a cytosolic Ca^2+^ increase-independent way [13], [14]. Many studies have reported additional mechanisms of posttranslational NOS-3 activation involving, for example, its phosphorylation in Ser^1177^ [15]. The produced NO causes vasorelaxation primarily by activating soluble guanylyl cyclase (sGC) in smooth muscle cells to increase intracellular cGMP, which in turn activates protein kinase G to induce vasorelaxation by decreasing cytosolic Ca^2+^ concentration ([Ca^2+^]_c_) [16], [1].

It has been demonstrated that the CNP-induced relaxation is partially mediated by the vascular endothelium in certain vascular beds [17], [18]. Given that CNP and NO are crucial regulators of vascular tone, several studies have highlighted the possibility that the vascular effect of CNP involves activation of NOS-3 followed by NO production. This activation would be mediated by the NPR-C receptor coupled to the Gi/PLC pathway through stimulation of Ca^2+^ influx [8], [9]. In this sense, Brunner and Wolkart [18] demonstrated that CNP induces relaxation of the rat coronary resistance vessels via NO-cGMP pathway. In addition, Murthy [19] showed that NOS-3 is activated by proteins coupled to NPR-C via stimulation of Ca^2+^ influx in gastrointestinal smooth muscle. Other authors have demonstrated that ANP and CNP interact with NPR-C receptor leading to an increase in NOS activity and NO production in cardiac ventricle and atria and aorta artery slices [20], [21]. However, the relationship between the activation of the NPR-C receptor by CNP and the effects of NO in conductance artery, where the endothelium-dependent relaxation is mainly attributed to NO, is not yet well established.

We hypothesized that the relaxation induced by CNP in rat isolated aorta involves the activation of endothelial NPR-C receptor with consequent NO production by distinct mechanisms of NOS-3 activation. Therefore, the aim of this study was to investigate the putative contribution of NO through NPR-C activation in the CNP-induced relaxation in isolated conductance artery.

## Materials and Methods

### 1. Animals

Male Wistar rats (180–250 g) were maintained under standard conditions, which included 12-h light/dark cycle and free access to both food (standard rat chow) and water. The pharmacological studies were performed in strict accordance with the Ethical Principles in Animal Research adopted by Brazilian College of Animal Experimentation. The protocol was approved by the Committee on the Ethics of Animal Experiments of the School of Medicine of Ribeirão Preto - University of São Paulo (CETEA Protocol n° 071/2009). All animals were anesthetized by inhalation of isoflurane (250 uL/250 g) prior to the decapitation thus preventing any suffering of these animals.

### 2. Vessel Preparation

The rats were anesthetized and killed by decapitation. The thoracic aorta was quickly removed, dissected and cut into 4-mm-long rings. In some rings, the endothelium was mechanically removed by gently rolling the lumen of the vessel on a thin wire. The aortic rings were placed between two stainless steel stirrups and connected to an isometric force transducer (Letica Scientific Instruments, Barcelona-Spain) in order to measure the isometric tension. The rings were placed in a 10 mL organ chamber containing Krebs solution with the following composition (mmol/L): NaCl 130, KCl 4.7, KH_2_PO_4_ 1.2, MgSO_4_ 1.2, NaHCO_3_ 14.9, glucose 5.5, and CaCl_2_ 1.6. The solution was maintained at pH 7.4 and gassed with 95% O_2_ and 5% CO_2_, at 37°C.

### 3. Functional Studies

The aortic rings were initially stretched to a basal tension of 1.5 g and allowed to equilibrate for 60 min in the bath, being washed every 15 min. In order to ensure the vascular smooth muscle functionality, the contractile response induced by 60 mmol/L KCl was tested in the resting tension of 0.5 g to 2.5 g. In our hands, the best response was obtained in 1.5 g that was standardized as the resting tension for the vascular reactivity studies. Furthermore, the aortic rings relaxed 100% in response to sodium nitroprusside and other NO donors used. Then, the aortic rings were continuously stimulated with phenylephrine 0.1 µmol/L, which is the concentration that produces half-maximal contraction (EC_50_), until reproducible contractile responses were obtained. Endothelial integrity was qualitatively assessed by the degree of relaxation elicited by acetylcholine (1 µmol/L) in the presence of the contractile tone induced by phenylephrine. For studies of endothelium-intact vessels, the ring was discarded if relaxation with acetylcholine was not 80% or greater. For studies of endothelium-denuded vessels, the rings were discarded if there was any degree of relaxation. The concentrations of acetylcholine and phenylephrine were selected on the basis of previous studies conducted in our laboratory.

After the equilibration period, specific protocols were performed on rat aortic rings. The aortic rings were pre-contracted with phenylephrine (0.1 µmol/L) and on top of the contractile response cumulative concentration-effect curves to CNP (0.01 nmol/L –0.5 µmol/L) were constructed in the absence or presence of the following drugs. In order to study the effect of the inhibitors, the arterial rings were incubated for 30 min with: *N^G^*-nitro-_L_-arginine methyl ester (_L_-NAME) (a non-selective NOS inhibitor 100 µmol/L) or hydroxocobalamin (an intracellular NO^0^ scavenger 10 µmol/L). The concentration-effect curves induced by CNP were compared to the control curves that were constructed in the absence of these drugs.

### 4. Confocal Microscopy and Image Analysis

The thoracic aortas were isolated and dissected. Cross sections of aortic rings (100 µm thick) with endothelium were placed vertically on glass coverslips covered with poly-l-lysine, in Ca^2+^ free Hanks solution with the following composition in mmol/L: 145.0 NaCl, 5.0 KCl, 1.0 MgCl_2_, 0.5 NaH_2_PO_4_, 10.0 dextrose and 10.0 HEPES at pH 7.4. The slice preparations were kept in a humidified 37°C incubator gassed with 5% CO_2_. To assess [Ca^2+^]_c_, slice preparations were loaded with the fluorescent Ca^2+^ dye Fluo-3 AM (10 µmol/L) for 30 min at room temperature, in Hanks solution containing 1.6 mmol/L CaCl_2_, at pH 7.4. Excess of dye was removed by washing out the dye with Hanks solution and allowing 30 min for intracellular desterification of Fluo-3 AM. Slice preparations were imaged in Hanks buffer (pH 7.4). [Ca^2+^]_c_ was assessed by Fluo-3 AM fluorescence with a confocal scanning laser microscope (Leica TCS-SP5). It was excited with the 488 nm line of an argon ion laser, and the emitted fluorescence was measured at 510 nm. A time-course software was used to capture images of the cells at 1.014 s intervals (xyt), in the Live Data Mode acquisition at 1024×1024 pixel at 700 Hz. Using the LSCM computer software, the intensity of the intracellular maximum or minimum fluorescence was measured in the endothelial and the smooth muscle sliced regions of interest, before and after addition of CNP at the concentration considered able to induce the maximal relaxant effect (ME) reached in concentration-effect curves (EC_100_∶0.3 µmol/L). Control responses were obtained in experiments where the vehicle (deionized water) was added instead of CNP. The initial fluorescence intensity value was obtained at t = 0 and it was designated F_0_ and the final fluorescence intensity value obtained after stimulation with CNP or vehicle was designated F. In this way, the percentage of the difference in fluorescence intensity (%ΔFI), which reflects the [Ca^2+^]_c_ increase in endothelial cells and the decrease in smooth muscle cells, were obtained in relation to F_0_ (100%). It was calculated by the following formula: %ΔFI = (F–F_0_/F_0_)×100.

### 5. Western Blot Analysis

The thoracic aortas were collected, dissected and cut into 4-mm long rings as previously described for the confocal analysis. Then the aortic rings with endothelium were submitted to the same procedures previously described for functional studies except that after the equilibration period, the aortic rings were pre-contracted with phenylephrine (0.1 µmol/L) and on top of the contractile response the vehicle was added or CNP 0.3 µmol/L. After CNP reached its maximum effect, rings were immediately frozen in liquid nitrogen. Each sample was homogenized in modified RIPA buffer (Tris-HCl 65.2 mmol/L; NaCl 154 mmol/L; NP-40 1% sodium deoxycolate 0.25%; EDTA 0.8 mmol/L; PMSF 1 mmol/L; Sodium orthovanadate 10 mmol/L; Sodium fluoride 100 mmol/L; Sodium Pyrophosphate 10 mmol/L and protease inhibitor) to prevent proteolysis and maintain the phosphorylation of proteins. Homogenates were centrifuged at 10.000 rpm and 4°C for 10 min to remove tissue debris. Protein concentrations in the samples were determined by Bradford method [22] (Quick Start Bradford, Bio-Rad). Protein from the tissue samples (30 µg) were separated on 8% SDS-PAGE and transferred to a nitrocellulose membrane. Membranes were blocked for 1 h with 5% nonfat milk in *Tris*-buffered solution at room temperature. Membranes were incubated with rabbit primary antibody anti-NOS-3 Ser^1177^ (1∶2000, Cell signaling) overnight, at 4°C. Afterwards, membranes were incubated with a HRP-conjugated mouse anti-rabbit secondary antibody (1∶1000, Santa Cruz Technology) for 1 h at room temperature. Protein bands were identified by means of chemiluminescence (ECL plus, GE Healthcare) and measured by densitometry. Expression levels of p-NOS-3 were normalized by total NOS-3.

### 6. NPR-C-receptor Expression by Immunohistochemistry

The animals were anesthetized and perfused with a buffer of 10% paraformaldehyde. The segments of isolated arteries were removed from the rats and fixed in formalin for 24 h. After this period, the segments were washed in tap water and kept in 70% alcohol. Thereafter, the artery segments were dehydrated, embedded in paraffin, cut on a microtome (4-µm thick) and mounted on glass slides coated with poly-L-lysine. The sections were deparaffinized, rehydrated and immersed in 10 mmol/L citrate-buffer at pH 6.0 and then submitted to the recovery of heat-induced epitope (HIER) by using steam for 45 min. Then the slides were washed with phosphate buffered saline (PBS) and soaked in 3% hydrogen peroxide for 20 min to block endogenous peroxidase. The links between nonspecific proteins were blocked by incubation for 30 min with normal serum (Vectastain ABC Elite Kit, Vector Lab). Then, the slides were incubated with primary antibody for detection of NPR-C-receptor (1∶100) (ABCAM, Cambridge, MA, USA) at 25°C for 2 h in a moist chamber. After washing with PBS, the slides were incubated with secondary biotinylated antibody (Vectastain ABC Elite Kit, Vector) for 30 min. They were then treated with avidin-biotin-peroxidase for more than 30 min and developed with red chromogen NovaRED Kit (Vector Lab) for 5 min. The counter-coloration was carried out with Harris Hematoxylin (Biomeda). Immunostaining was considered positive when the cell membrane was homogeneously stained in red. As negative controls, all specimens were incubated with an isotope-matched control antibody under identical conditions.

### 7. Statistical Analysis

Data are expressed as mean ± S.E.M. In each set of experiments, *n* indicates the number of rats used. The values of vascular reactivity responses to CNP are expressed as percentage of the preceding contraction induced by phenylephrine. The concentration of the agonist producing a half-maximal response (EC_50_) was determined after *logit* transformation of the normalized concentration-response curves, and it is reported as the negative logarithm (−log EC_50_ = pD_2_ values) of the mean of individual values for each tissue. The maximal relaxant effect (ME) was considered to be the maximal amplitude response reached in concentration-effect curves to CNP. Student *t* test was used to assess statistical differences in the Western blotting experiments. In confocal microscopy experiments, the decrease or increase in [Ca^2+^]_c_ in aortic rings with endothelium stimulated with CNP was obtained from %Δ FI. Statistical significance was tested by unpaired Student *t* test. Values of *, p<0.05, **, p<0.01, and ***, p<0.001 were considered to be significant.

### 8. Drugs

Acetylcholine, phenylephrine, C-type natriuretic peptide, hydroxocobalamin and FLUO-3AM were purchased from Sigma Chemical Co. (St. Louis, MO-USA). Acetylcholine, phenylephrine and hydroxocobalamin were diluted in deionized water. C-type natriuretic peptide was dissolved in acetic acid solution 5% and diluted in deionized water. FLUO-3AM was prepared in Hanks physiological solution.

## Results

### 1. Relaxation Induced by CNP in Intact and Denuded Endothelium Rat Aorta after Contraction with Phenylephrine

**Figure 1:**
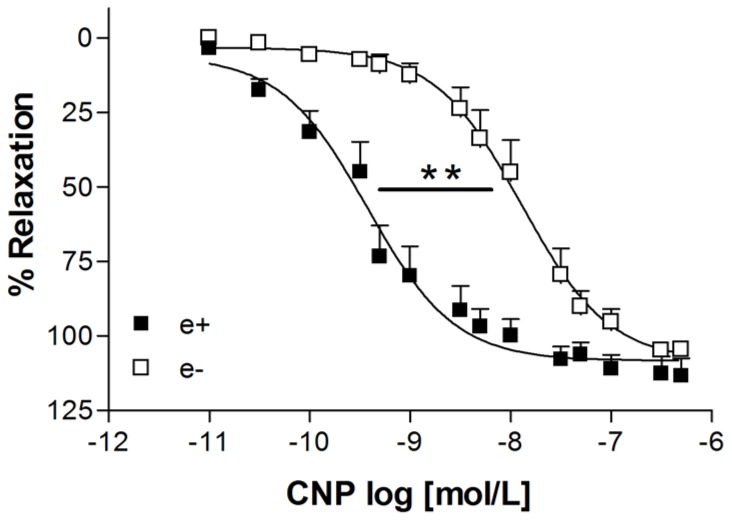
Relaxation induced by CNP in intact endothelium (e^+^) and denuded endothelium (e^−^) aortic rings after contraction with phenylephrine. Responses are represented as the percentage of the relaxation induced by CNP on the contraction with phenylephrine (0.1 µmol/L). Data are means ± SEM of at least five experiments. **P<0.01 for *p*D_2_ values were obtained for e^−^ *vs.* e^+^ rat aortic rings (Student *t* test).

**Figure 2:**
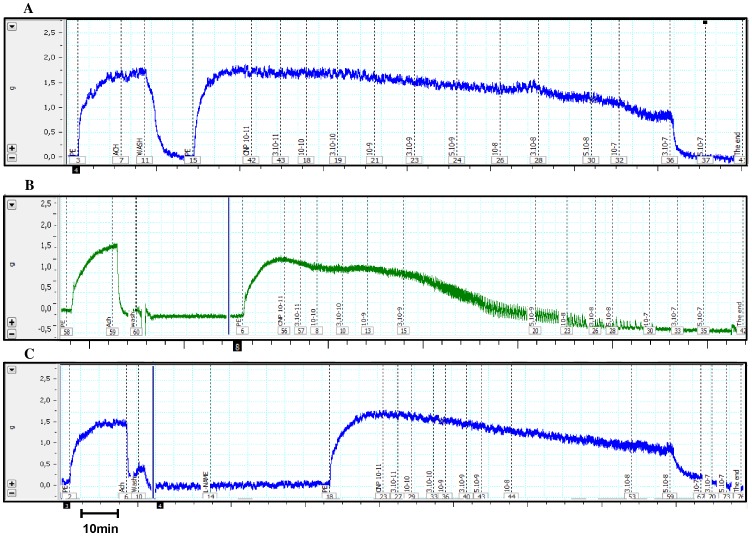
Representative tracings showing the amplitude of the pre-contraction induced by phenylephrine and the profile of relaxation induced by CNP. A) In endothelium-denuded aortic rings B) Endothelium-intact aortic rings, C) Endothelium-intact aortic rings after incubation with L-NAME.

The cumulative addition of CNP to the organ bath solution during the sustained contraction induced by phenylephrine was able to promote concentration-dependent relaxation with similar maximum effect in intact endothelium (ME: 113.4±6.0%, *n* = 5) and denuded endothelium aortic rings (ME: 104.6±2.2%, *n* = 9). However, in denuded endothelium aortic rings the relaxation induced by CNP was less potent as shown by the pD_2_ values (7.90±0.16, *n* = 9) than in intact endothelium aortic rings (10.74±0.95, *n* = 5) (Fig. 1). As shown in the Fig. 2, the contractile response induced by phenylephrine was not significantly different in denuded aorta (1.87 g ±0.22, n = 9) and endothelium-intact aortas (1.45 g ±0 16, n = 5, P = 0.16). This response was not different between the data obtained after incubation with the NO-synthase inhibitor L-NAME (1.93 g ±0.16, n = 6, P = 0.07).

### 2. Effects of L-NAME and Hydroxocobalamin on the Relaxation Induced by CNP in Intact Endothelium Aortic Rings

**Figure 3:**
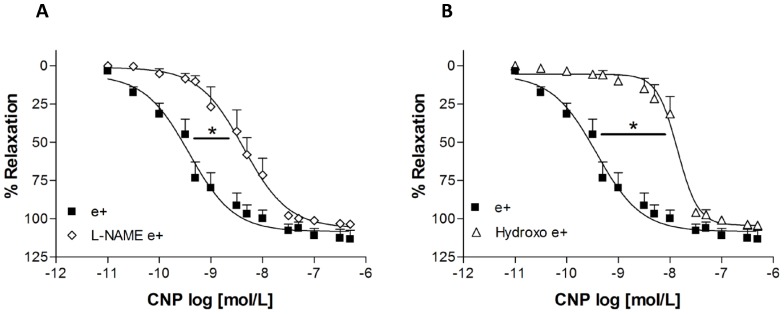
Effects of L-NAME and hydroxocobalamin on the relaxation induced by CNP in intact endothelium aortic rings (e^+^). Concentration-effect curves were constructed in the absence (control) or after incubation with these drugs for 30 minutes, _L_-NAME (100 µmol/L) (A) and hydroxocobalamin (Hydroxo, 10 µmol/L) (B). Responses are represented as the percentage of the relaxation induced by CNP on the contraction with phenylephrine (0.1 µmol/L). Data are means ± SEM of five experiments. *P<0.05 for pD_2_ values was obtained with _L_-NAME or hydroxo *vs.* intact endothelium rat aortic rings (e^+^) (Student *t t*est).

Incubation with the NOS inhibitor, L-NAME, or the NO scavenger, hydroxocobalamin, did not alter the contractile response induced by phenylephrine. The maximum relaxation induced by CNP was not altered by L-NAME (in the presence: 103.6±1.6%, n = 6 and in the absence of L-NAME: 113.4±6.0%, n = 5). However, L-NAME significantly attenuated the potency of CNP (pD_2_∶8.40±0.20, n = 6) when compared to CNP-relaxation in the absence of L-NAME (pD_2_∶10.74±0.95, n = 5) (Fig. 3A). Similar results were obtained in the presence of hydroxocobalamin. The maximum effect was not altered by hydroxocobalamin (ME: 104.3±1.9%, n = 5) when compared with the maximum effect observed with CNP in the absence of hydroxocobalamin (ME = 113.4±6.0%, n = 5). On the other hand, hydroxocobalamin attenuated the potency of the CNP in intact endothelium aortic rings (pD_2_ in the presence of hydroxocobalamin: 7.90±0.09, n = 5) (Fig. 3B). In fact, similar values of potency for CNP were obtained in aortic rings with intact endothelium in the presence of L-NAME or hydroxocobalamin and in denuded endothelium aortic rings (open symbol in the Fig. 1 and Fig. 3A and Fig. 3B).

### 3. NOS-3 Phosphorylation

**Figure 4:**
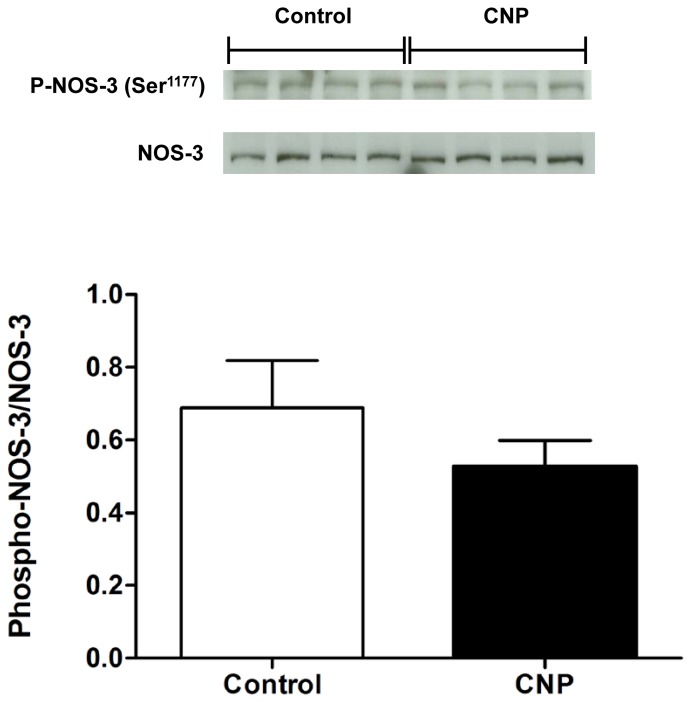
Phosphorylation of NOS-3 in rat aorta. Representative blots showing the protein expression of p-NOS-3-Ser^1177^ after stimulation with vehicle (control) and CNP (0.3 µmol/L). Each lane represents the protein obtained from the homogenate of an independent rat aorta. Bars show the optic densitometry of the blots (*n* = 6–7/group).

We analyzed the phosphorylation state on Ser^1177^ site of NOS-3 by Western blot. CNP did not change the phosphorylation of the activation site of NOS-3, Ser^1177^, when compared with the control in the absence of CNP (Fig. 4).

### 4. Cytosolic Ca^2+^ Increase in Endothelial Cells and Cytosolic Ca^2+^ Decrease in Smooth Muscle Cells Induced by CNP

**Figure 5:**
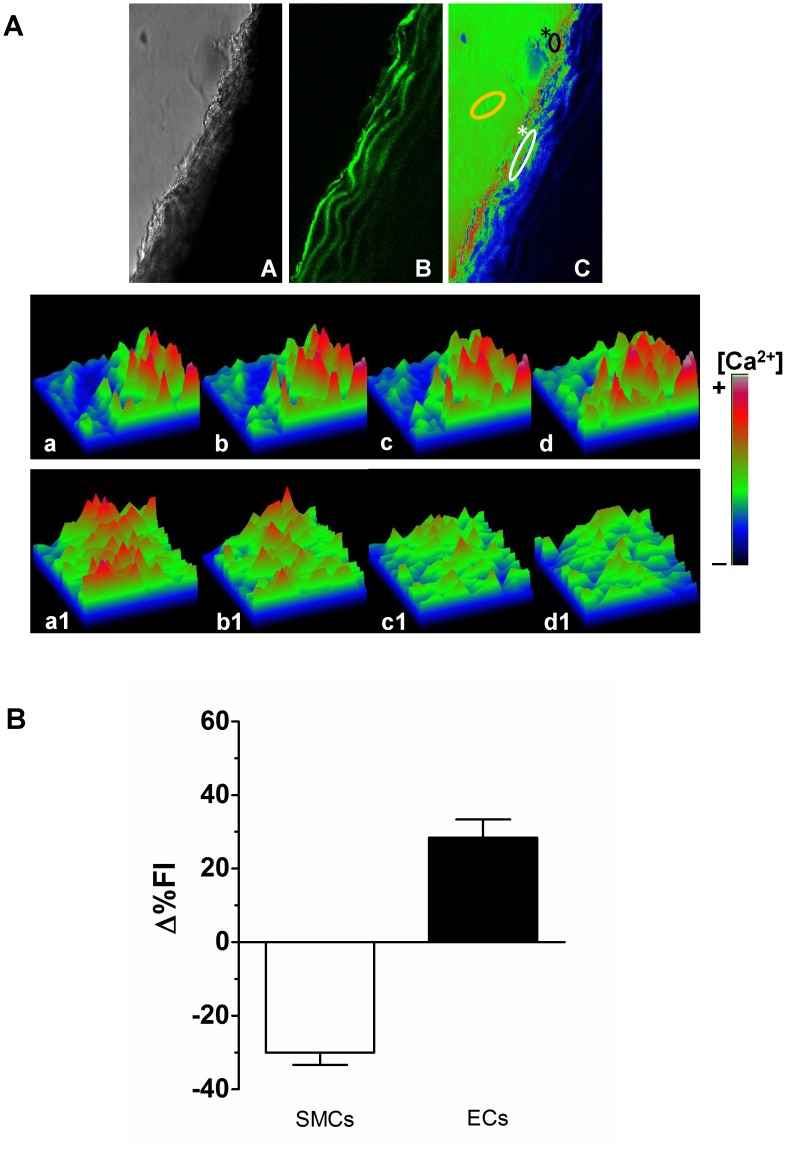
Cytosolic Ca^2+^ increase in endothelial cells and cytosolic Ca^2+^ decrease in smooth muscle cells induced by CNP. A) Aortic rings were preloaded with Fluo-3 AM and then stimulated with CNP (0.3 µmol/L). Serial Ca^2+^ images of Fluo-3 fluorescence in aortic segment were recorded at the times (t) 0, 210, 410 and 610 seconds (s) after addition of CNP. A) image in differential contrast phase-DIC; B) image of Fluo-3 fluorescence and C) merged image in pseudocolor (*black line ECs), (*white line SMCs) and (*yellow line arterial lumen). The effects of CNP were represented in surface plots of the endothelial cells (a–d) and the smooth muscle cells (a1–d1). The arbitrary intensity values from low to high [Ca^2+^] are indicated by pseudocolor values. B) Effect of CNP (0.3 µmol/L) on average fluorescence intensity (%ΔFI) of smooth muscle cells (SMCs) and endothelial cells (ECs) from aortic ring segment preloaded with Fluo-3 AM.

The fluorescence images at 488 nm were taken at confocal planes adjusted at nearly 0 and 10 µm from the bottom of the tissue. In such images of the aorta in the plane at 10 µm, both endothelial cells and vascular smooth muscle cells were simultaneously observed, and they could be readily distinguished from each other by their morphological differences. A major advantage of using the rat aorta artery segment was that Ca^2+^- images of both vascular smooth muscle cells and endothelial cells were obtained simultaneously at one confocal plane. The cross section of rat aorta artery segments was observed by an optical microscope and with Fluo-3AM. Figure 5 shows Ca^2+^-images of endothelial cells and vascular smooth muscle cells in rat aorta artery segment during the response to CNP that were selected and shown in pseudocolor. Changes in [Ca^2+^]_c_ in representative endothelial cells (* – black line) and vascular smooth muscle cells (* – white line) were plotted as surface plot. Figure 5A (a–d) shows the surface plot profile obtained from a delimited area in endothelial cells during the time-scan in the response to CNP. Figure 5A (a1–d1) represents the surface plot of limited area of vascular smooth muscle cells. As shown in the Figure 5A, we used the scale bar color (pseudocolor) in every single image. As shown in the Figure 5B, the addition of CNP (0.3 µM) produced an increase in [Ca^2+^]_c_ in endothelial cells (Δ%FI = 28.47±4.92%, n = 3) and a decrease in [Ca^2+^]_c_ in vascular smooth muscle cells (Δ%FI = −30.04±3.26%, n = 3).

### 5. Expression of NPR-C-receptors in Rat Aorta Artery

**Figure 6:**
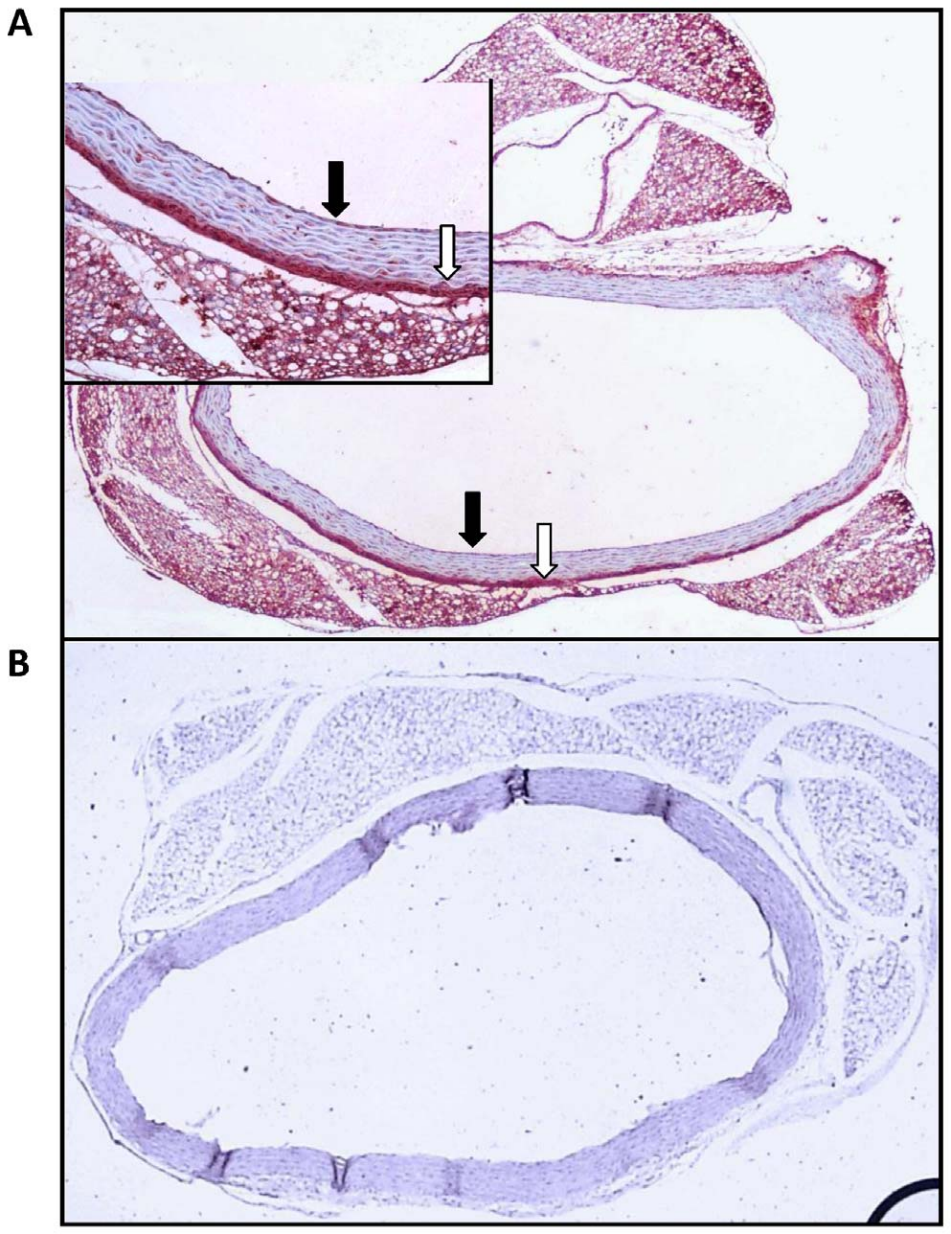
Expression of NPR-C-receptor in rat aorta artery. A) Representative photomicrography of an aortic ring showing endothelial (black arrows) and adventitial (white arrows) immunohistochemical staining for NPR-C receptor (×100, and inset ×400). B) Representative photomicrography of an aortic ring showing negative control to immunohistochemical staining for NPR-C receptor.

The presence of NPR-C-receptors was histologically analyzed in the cross-section preparations of the aortic rings, which contains the midregions of the arteries. Endothelial (black arrows) and adventitial (white arrows) staining for NPR-C-receptors were observed in rat aorta arteries. As shown in the Figure 6A, NPR-C receptors are localized in both endothelial cells and adventitial cells.

## Discussion

In the present study we demonstrated that the CNP-induced relaxation of aorta isolated from rats partially involves endothelial NO produced by NOS. Since pre-contraction with phenylephrine was similar in the presence and absence of endothelium, and also in intact-endothelium aortas after incubation with L-NAME, the same concentration of phenylephrine was used in all the vascular reactivity experiments. Removal of the aortic endothelium resulted in attenuation of the relaxant effect of CNP similar to that observed following blockade of NOS or intracellular NO scavenging. Furthermore, we showed that CNP is capable of increasing [Ca^2+^]_c_ in endothelial cells to activate NOS and produce NO. This NO released from endothelial cells by diffusing into vascular smooth muscle cells was able of decreasing [Ca^2+^]_c_ and to potentiate the CNP-induced aorta relaxation. Therefore, our findings are certainly relevant to the scenario of the cellular mechanisms involved in the NO pathway activation *during* the vascular relaxation induced by this peptide in intact conductance vessels.

Previous findings have demonstrated that the role of the endothelium in the CNP-induced vascular relaxation is controversial. It is known so far that CNP promotes relaxation in a manner independent of intact vascular endothelium in arteries [18], [23], [24] whereas in veins the endothelium appears to modulate negatively its relaxant effect [25]. In contrast to these studies, in the present work we observed that the CNP-induced relaxation of an intact conductance vessel, the aorta artery, involves an endothelial element since CNP was substantially less potent to promote relaxation in the absence of the endothelium. In this sense, similar results have been also shown by Brunner and Wolkart [18] in resistance arteries in which the endothelium was able to positively modulate the CNP-induced relaxation. Moreover, Sabrane et al. [26] demonstrated that the vascular endothelium is critically involved in the hypotensive and hypovolemic actions of ANP.

In this work, we provide consistent evidence that the endothelial element that positively modulate the CNP-induced relaxation is likely to be NO because the inhibition of NOS with _L_-NAME and the pre-incubation with an intracellular scavenger of NO resulted in attenuation that was similar to the relaxation observed in the absence of endothelium. The concentrations of L-NAME and hydroxocobalamin used in this work were also able to attenuate the vasorelaxant activity of the NO donor sodium nitroprusside in rat aorta [27] as well as acetylcholine [28]. Is well known, the production of NO in endothelial cells (ECs) is mainly generated through activation of endothelial NOS (NOS-3). ECs-derived NO subsequently activates sGC to elevate the production of cGMP and decrease [Ca^2+^]_c_ due to the activation of many proteins by phosphorylation resulting in vascular relaxation [29], [1]. Several groups have shown that peptides ANP and CNP are able to induce activation of NOS-3 in distinct preparations contributing to the vascular effects mediated by these peptides [30], [21], [31], [32]. However, in contrast to the other authors that showed that the blockade of NOS in isolated arteries did not affect [33] or potentiate [34] the CNP-induced relaxation. Although the exact reason for this discrepancy between our study and those ones is not clear, this may be due to differences in the species used in the experimental design adopted and/or in the vascular segments that were studied.

With regard to NOS-3 activation, it is known that such activation can be modulated through kinase-dependent signaling pathways that involve phosphorylation of the residues Thr^495^, Ser^615/617^, Ser^633/635^, or Ser^1177/1179^ that play an important role in the regulation of its enzymatic activity in ECs [35], [36], [37]. However, Ser^1177^ appears to be the most important among the NOS-3 phosphorylation sites because most stimuli that promote its activation are observed to cause phosphorylation of this site. Thus, the role of CNP on NOS-3 activation was evaluated by measuring NOS-3 phosphorylation at Ser^1177^. However, our results showed that phosphorylation of NOS-3 at activation residue Ser^1177^ did not change after stimulation with CNP leading us to suggest that NOS-3 activation mediated through kinase-dependent signaling pathways, more specifically the phosphorylation of the residue Ser^1177^, is not the pathway involved in the NO production that contribute to the CNP-induced relaxant effect in aorta isolated from rats. In this regard, we cannot rule out the possibility that phosphorylation may occur in other residues of NOS-3 that also has been described to be able to decrease (Thr^495497^) or increase (Ser^635/633^) the NOS-3 activity.

On the other hand, it is well established that the production of NO in endothelial cells by NOS-3 can also be Ca^2+^/calmodulin-dependent [38], [39]. It has been proposed that following a rise in [Ca^2+^]_c_ in endothelial cells, NOS-3 dissociates from caveolin-1, allowing activation of the enzyme [40]. However, with respect to the CNP-induced NOS-3 activation mode, it is not yet quite clear how this happens in intact conductance vessels *during* the vascular relaxation. Interestingly, we showed in the present study an increase in [Ca^2+^]_c_ in the endothelial cells followed by a decrease of [Ca^2+^]_c_ in the vascular smooth muscle cells after the administration of CNP. This result leads us to suggest that CNP induce that increased [Ca^2+^]_c_ in the endothelial cells could involve NO production since wich was inhibited by L-NAME and NO scavenger hydroxicobalamin. This CNP effect possibly occurs through a receptor-dependent mechanism. This notion is further supported by previous findings that showed that increased NOS activity induced by CNP, ANP and cANP (4–23) in aorta tissue was blunted by the inhibitor of calmodulin and the blockade of Ca^2+^ influx [21], [31] suggesting that the NOS activation induced by these peptides is mediated by a Ca^2+^/calmodulin-dependent mechanism.

As to the subtype of the natriuretic receptor involved in this mechanism, it has been demonstrated that NOS-3 is activated by G protein coupled to NPR-C in gastrointestinal smooth muscle and this effect is dependent on Ca^2+^ influx [30]. A similar conclusion was reached by other authors in rat coronary microvasculature [18], in aortic tissue [21], [31] and aorta artery from normotensive and spontaneously hypertensive rats stimulated with ANP [41]. Additionally, specific NPR-C receptor agonist, cANP (4–23), was capable of inducing an increase in NOS activity in aorta, heart and kidney [21]. Considering that the NPR-C activation triggers a signaling cascade that involves Ca^2+^ influx dependent on the activation of inhibitory G protein (Gi) and/or phospholipase C (PLC) [19], our data suggest that NPR-C receptor would be the receptor involved in NOS-3 activation induced by CNP in aorta isolated. Through stimulation of Ca^2+^ influx in endothelial cells, CNP would induce an activation of NOS-3 leading to NO release and consequently relaxation of the vascular smooth muscle cells. This hypothesis was best supported by our result with immunohistochemical staining demonstrating that the NPR-C receptor is expressed in endothelial cells of aorta isolated from rats although further studies are necessary to elucidate the involvement of this receptor from a functional standpoint.

## Conclusions

Our findings suggest that the CNP-induced relaxation in intact aorta isolated from rats involves NO production in endothelial cells. The major novel finding of this study was that in a conductance vessel the CNP-induced NO production is due to [Ca^2+^]_c_ increase in endothelial cells possibly through NPR-C activation expressed in these cells. These results may be construed as an important step in the understanding of the possible cross-talk between CNP and NO.
